# Cancer in indigenous people in Latin America and the Caribbean: a review

**DOI:** 10.1002/cam4.134

**Published:** 2013-12-03

**Authors:** Suzanne P Moore, David Forman, Marion Piñeros, Sdenka M Fernández, Marceli Oliveira Santos, Freddie Bray

**Affiliations:** 1 International Agency for Research on CancerLyon, France; 2 Instituto Nacional de CancerologíaBogotá, Colombia; 3 Regitro de Cáncer de la PazLa PAz, Bolivia; 4 Instituto Nacional de Câncer José Alencar Gomes da Silva – INCA/MSRio de Janeiro, Brasil

**Keywords:** Cancer, cancer registration, disparity, indigenous, Latin America and the Caribbean, systematic review

## Abstract

Cancer is a leading cause of death in Latin America but there have been few assessments of the cancer burden for the 10% of the population who are indigenous. Evidence from other world regions suggests cancer survival is poorer for indigenous people than for others due to a greater incidence of case-fatal cancers, later stage at diagnosis, and less cancer treatment. A status report on the cancer profile of indigenous people in Latin America and the Caribbean (LAC) is therefore clearly warranted. We undertook a systematic review of the peer-reviewed literature in academic databases, and considered evidence from cancer registries from 1980, to assess cancer epidemiology among indigenous people in LAC. We identified 35 peer-reviewed articles pertaining to cancer in indigenous people. Rates of cervical cancer in parts of Brazil, Ecuador, and Guyana, stomach cancer rates in regions of Chile and gallbladder rates in Chile and Bolivia, were higher for indigenous compared to others. Breast cancer rates were lower in Ecuador, Brazil, and Chile. Six cancer registries in Brazil provided incidence data but no other reports of incidence, mortality, or survival were identified. There was a paucity of data surrounding the cancer burden of indigenous people in LAC. In view of predicted increases in cancer rates in ensuing decades, and the disparities in burden already experienced by indigenous people in the region, it is imperative that cancer profiles are obtained and cancer control measures identified and prioritized.

## Introduction

Cancer is a leading cause of death in Latin America and the Caribbean (LAC) 1 but for the 10% of the population who are indigenous 2, little attention has been given to measuring their cancer burden, and elucidating underlying cancer determinants and access to prevention and treatment services. Indigenous peoples, who are culturally heterogeneous across countries and within countries in the region, are often marginalized and without political representation 3,4 and have disproportionally worse health than their nonindigenous counterparts 5. A number of countries with well-resourced cancer registries, such as Australia and New Zealand, report that cancer is now the second leading cause of death among indigenous peoples 6,7, and that survival is lower for indigenous compared to nonindigenous people 8. Factors contributing to poorer survival include greater incidence of case-fatal cancers, later stage at diagnosis, and less cancer treatment 9, but the extent of these prognostic factors among indigenous people in Latin America in not known. In addition, poverty has been strongly associated with poor cancer outcomes 10 and indigenous people are among the poorest people in LAC 11. A population-based situation analysis of cancer burden among indigenous people in the region is therefore clearly warranted.

A recent comprehensive review of cancer care in Latin America by The Lancet Oncology Commission highlighted the lack of data on cancer outcomes among indigenous populations 12. On the basis of a systematic review of the literature, this study aims to provide a status report on the cancer profile among indigenous people within LAC, and identify key areas where information is sparse, and for which improved surveillance would provide a greater understanding of the cancer burden and the causes of cancer disparities among indigenous populations.

## Methods

A formal definition of indigenous peoples has not been adopted by the United Nations, the diversity of indigenous populations rendering definitions problematic 13. Rather, a modern and inclusive understanding of “indigenous” has been developed and includes peoples who identify and are identified as indigenous, demonstrate historical continuity with precolonial societies, have distinct social, economic, or political systems, maintain distinct languages, cultures, and beliefs, and form nondominant groups of society 14. In this review, we document how indigenous status is determined in each LAC country, assess which countries have population-based cancer registries, and whether indigenous status is recorded therein.

Literature searches were undertaken using PubMed and databases pertinent to the region, including LILACS, SciElo, Biblioteca-CEPAL. Key terms used were “indigenous,” “Indian,” “Amerindian,” together with “neoplasm” and “cancer,” with specific searches carried out on the basis of identified names of indigenous populations. English, Portuguese, and Spanish language publications, after 1980, were included. Electronic searches for/of cancer registry or government reports, as well as scrutiny of hard copy reports, were conducted. Where information on indigenous status was lacking, but data were available for regions with a predominantly indigenous population, we utilized available cancer rates.

Age-adjusted incidence rates per 100,000, available for indigenous people from six population-based cancer registries (PBCR) in Brazil, were compared with corresponding rates for the whole population. Rates were calculated by age, sex, and cancer to the world population. Population data were obtained from the 2010 Brazilian Census 15.

## Results

The literature review yielded 1794 articles, 35 of which were included. No cancer rates for indigenous people were found in cancer registry reports, and contacts at registries in Argentina, Bolivia, Colombia, Guatemala, Ecuador, Mexico, and Peru confirmed either that these data were not routinely collected or that reports did not exist; incidence data were made available from six registries in Brazil.

### Demography of indigenous populations in LAC

**Figure 1:**
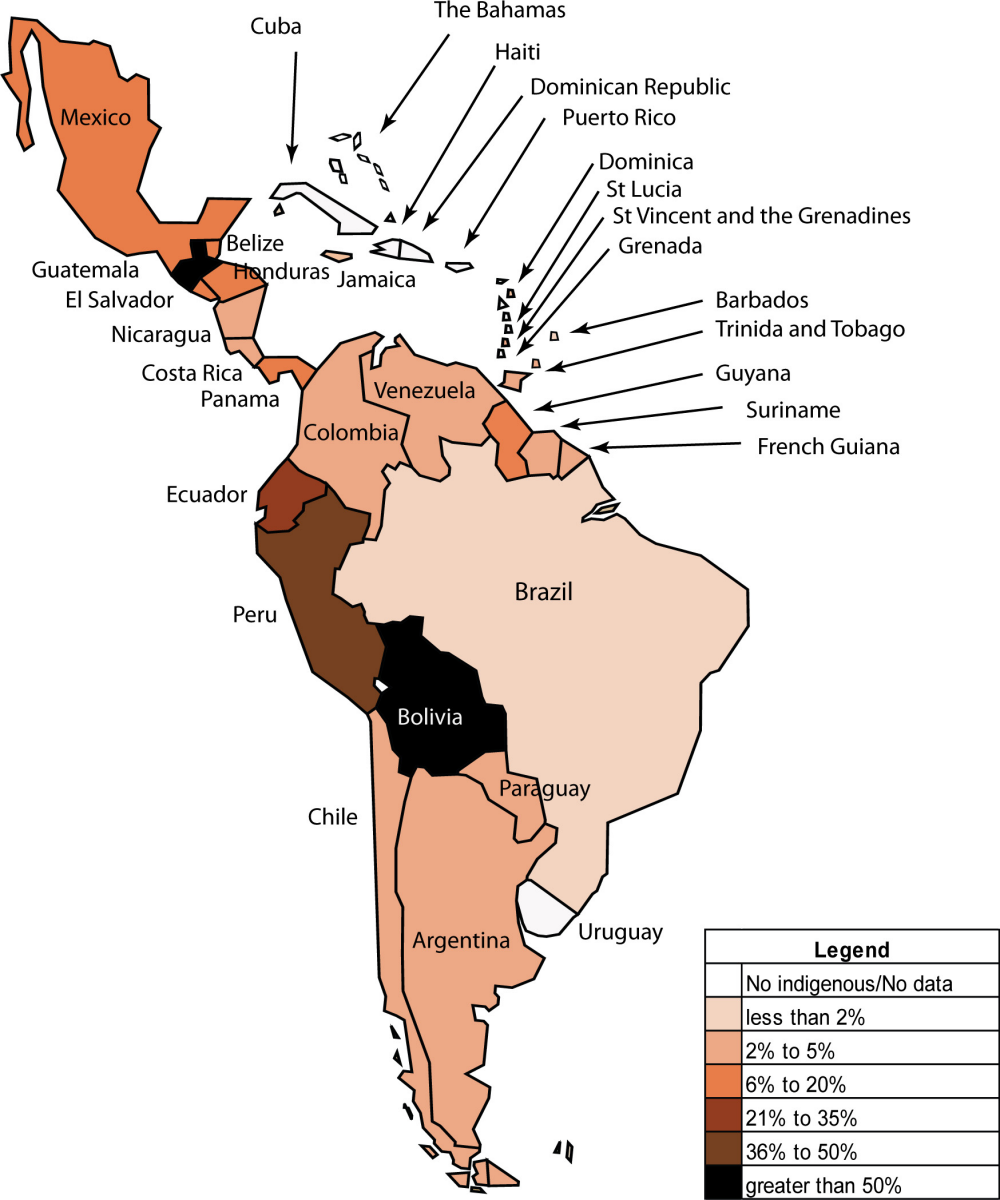
Percentage of indigenous people in Latin America and the Caribbean 5,17,45,91.

There are over 400 indigenous groups in LAC, encompassing 45–48 million people 5, ∼90% of whom live in five countries, namely Bolivia, Guatemala, Peru, Ecuador, and Mexico 16. In Bolivia and Guatemala, indigenous peoples represent the majority of the population, 62% and 60%, respectively 17, whereas in Brazil, Paraguay, and Venezuela, the indigenous population comprise less than 3% of the total population 5,17 (Fig. 1). There is discrepancy regarding the proportion of indigenous people in El Salvador and Mexico 5,18.

“Indigeneity” is determined variously throughout the region (Table 1) and not all indigenous people are included in national statistics, for example, where fear of discrimination may deterrent them from self-identifying as indigenous, or census surveys do not include a particular district 17. A 2005 report by the Economic Commission for Latin America (Spanish acronym CEPAL) provides the following criteria for indigenous identity in LAC: common ancestry, attachment to culture, and development of consciousness 19.

**Table 1:** Indigenous status, cancer registration availability, and recording of indigenous status by registry

Country	How is indigenous status defined?	Cancer registration^1^	Registry records indigenous status
South America			
Argentina	Based on self-identification or self-ascription 93	Yes	No
Bolivia	Self-identification 75	Yes	No
Brazil	Self-identification by phenotype: combines skin color, physical characteristics, region 76. Includes *branco* (white), *preto* (black), *pardo* (malutto or brown), *indegena* (indigenous), *amerela* (yellow)	Yes	Yes
Chile	Self-declared 38	Yes	Not clear
Colombia	Self-identification 75	Yes	No
Ecuador	Speaks an indigenous language 94	Yes	No
French Guiana	NA	Yes	No
Guyana	Self-identification 75	Yes	Yes
Paraguay	Self-identification 75	Yes	No
Peru	Indigenous mother tongue 75	Yes	No
Suriname	Indigenous people not recognized 17	Yes	No
Venezuela	NA	Yes	No
Uruguay	No indigenous people 75	Yes	No
Central America			
Belize	Self-identification 75	Yes	No
Costa Rica	NA	Yes	No
El Salvador	NA	Yes	No
Guatemala	Self-identification 75	Yes^2^	Yes
Honduras	NA	Yes	No
Mexico	Speaks indigenous language 75	Yes	No
Nicaragua	Indigenous mother tongue 75	Yes	No
Panama	Self-identification 75	Yes	No
Caribbean^3^			
Cuba	NA	Yes	No
Dominica	NA	No	No
Dominican Republic	NA	No	No
Grenada	NA	Yes	No
Saint Vincent & the Grenadines	NA	No	No

1 Data from IARC records; refers to population-based cancer registration.

2 Not population based.

3 Caribbean counties with no evidence of indigenous populations: Antigua & Barbuda, Barbados, The Bahamas, Guadelope, Haiti, Jamaica, Martinique, Netherland Antilles, Puerto Rico, Saint Kitts & Nevis, Saint Lucia, Trinidad and Tobago.

All Latin American countries are classified by the World Bank as low or middle income countries 20 and the World Bank reports that being indigenous increases an individual's probability of living in poverty, and having less education and access to health care 11. In addition, indigenous people in LAC have reportedly higher rates of premature mortality and morbidity than nonindigenous counterparts 16. The disproportion in poverty between indigenous and others has been reported in both Ecuador 21 and Guatemala 17, already among the poorest countries in Latin America.

### Availability of routine cancer data in LAC

In Central and South America, 13% of the population is covered by population-based cancer registration and 95% of the population by a national mortality scheme 22. There is at least one population-based cancer registry in each country (Table 1) while seven of the 13 countries in the Caribbean have cancer registration schemes 23,24. To the best of our knowledge indigenous status is currently recorded in registries of only four countries, Brazil, Guyana, Guatemala, and Chile (Table 1).

### Burden of cancer in indigenous people in LAC

To date there has been no regional overview of cancer among indigenous people in LAC. Country-specific searches identified 26 articles with reference to cancer among indigenous people, the majority relating to cervical cancer (*n* = 11) and nine additional articles report cancer risk factors. Below, we describe the limited information on rates and cancer profile among indigenous peoples within LAC.

#### Bolivia

In Bolivia, where the majority are indigenous, cervical cancer was the most commonly occurring cancer in women, and the leading cause of cancer death 25, the estimated incidence and mortality rates (2008) higher than in other countries in South America 26. Rates of gallbladder cancer in La Paz (1978–1980) among Mestizo and Indian people were two to three times higher, respectively, than for the white population 27, but there is no recent report.

#### Brazil

A report on mortality among indigenous people in the Rio Grande do Sul region of Brazil cites lung cancer as the third, cervical cancer the seventh, and stomach cancer the ninth leading causes of death 28. Cancer, predominantly of the cervix, was identified as the fourth most common cause of death in the Suya people of the Xingu Indian Park from 1970 to 2004 29 and high incidence of cervical cancer was reported among indigenous women in the Park 30, but no rates were available. No cases of breast cancer were reported among Indian women in the Terena region 31 and southern Brazilians with European heritage were reportedly more at risk of melanoma compared to those of indigenous ancestry 32.

**Figure 2:**
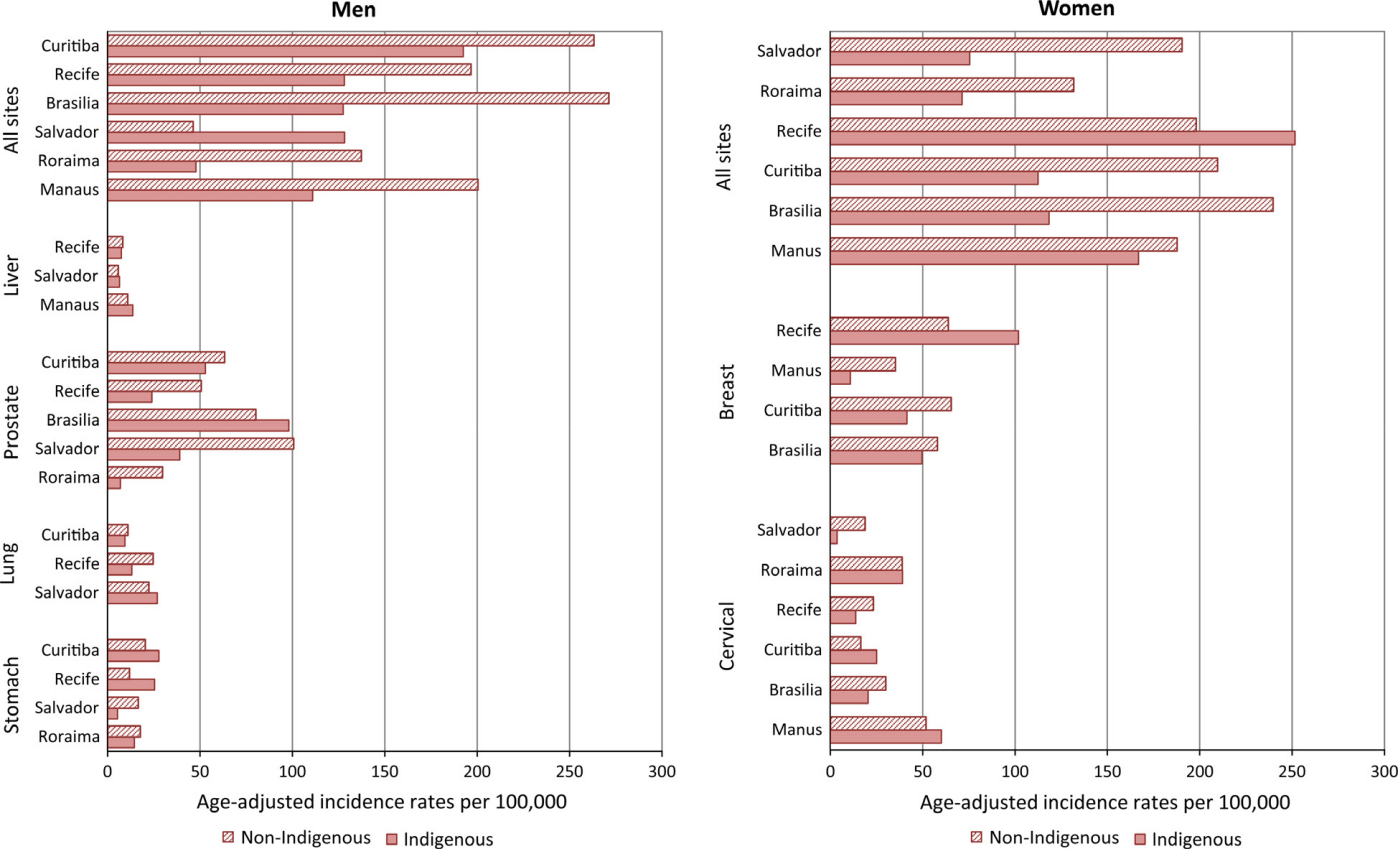
Age-adjusted incidence rates per 100,000 for indigenous versus nonindigenous people for five Brazilian cities: Recife (1995–2005), Salvador (1996–2005), Manaus (1999–2005), Curitiba (1998–2007), Brasilia (1999–2002), and the state of Roraima (2003).

Indigenous people make up ∼0.2% of the population in Recife, Manaus, Curitiba, and Brasilia, 3% in Salvador, and 11% in the state of Romaima, five cities and one state for which cancer incidence rates were available from the PBCR (Fig. 2) 33. The total number of cases was small and varied considerably between registries, as did the periods covered, rendering comparability difficult. Prostate cancer was most commonly occurring among men in Salvador, Brasilia, and Curitiba, as was stomach cancer in Roraima, Recife, and Curitiba. Cervical cancer was most common among women in Roraima and Manaus, the second in Brasilia and Curitiba, and the third in Recife and Salvador. Proportions of incident cancers among indigenous and nonindigenous people in Roraima were similar (0.03% of both populations) (data not shown). When compared with the whole population, age-adjusted incidence rates were less for the most commonly occurring cancer types among indigenous people in all six locations, with the exception of all sites in men in Salvador (Fig. 2).

#### Chile

A study of oral cancers in the Tumuco region (1994–2008) detected differences in people whose name indicated Mapuche descent 34. Greater incidence of stomach cancer was reported among women (but not men) of Mapuche descent compared to other groups (RR: 2.2; 95% CI: 1.2–3.7) 35 and similar sex disparity was reported in a study of gallbladder cancer 36. A further study of gallbladder cancer found an increased risk of the disease for those of Mapuche descent and, as American Indians have similarly high rates of gallbladder cancer 37, the authors suggest genetic predisposition as a determinant in both populations 38. Greater mortality from gallbladder cancer has also been reported in rural areas of Chile, particularly for those of Mapuche descent 39. Aymara women were reported to have a lower incidence of breast cancer in the Arica region of Chile, but the paper provided no rates 40.

#### Colombia

There are no rates available for indigenous people in Colombia, however, estimates in the La Guajira region, where 45% are indigenous, found lower cancer rates than Colombia overall (62/100,000 vs. 167/100,000, respectively) 41. The risk of dying from cervical cancer was highest among women from the Amazon region 42, and stomach cancer mortality rates were highest in the Cauca region 43, both regions with a very high indigenous population.

#### Ecuador

Standardized breast and cervical cancer rates, primarily among indigenous women in four regions of the Amazon Basin of Ecuador, were 5.08 and 21.58 per 100,000, respectively, for the period 1985–1998. Rates of breast cancer were less than those of the whole of Ecuador (30.8/100,000) and more developed countries (66.4/100,000) but cervical cancer rates were similar to those of all women in Ecuador (27.1/100,0000) and considerably higher than those of women in developed countries 44. Cancer incidence among indigenous compared to nonindigenous people of the Ecuadorian Amazon Basin, other than for melanoma, was similar for the period 1985–2000 45.

#### Guyana

Cervical cancer was more common among Indian women than other groups, and significantly associated with higher prevalence of human papillomavirus (HPV) infection, earlier age at first intercourse, greater number of births, and lower socioeconomic status 46. A study of cervical cancer and HPV in 2250 Guyanese indigenous women found that 18 women (0.8%) had invasive cervical cancer 47.

#### Peru

Two case studies in Peru reported Kaposi's sarcoma in three HIV-negative Quechua men 48,49.

#### Overall cancer profile in LAC

The most commonly occurring cancers in Latin America overall are prostate, lung, stomach, colorectal, and bladder in men and breast, cervix, colorectal, stomach, and lung in women 50. As there are no summary tables available, we are unable to report the most common among indigenous peoples. However, the limited evidence above suggests that most commonly occurring in some indigenous populations are of the cervix, stomach, prostate, and gallbladder.

### Risk factors for cancer in indigenous people in LAC

Prevalence of cancer risk factors among indigenous people was reported in nine articles. Higher smoking rates were reported among indigenous people in the Southern Andes of Argentina than those of European descent 51 and changing alcohol use and subsequent alcohol abuse was reported in Indian people in Venezuela 52,53, but cancer risk is not mentioned.

Human T-cell lymphotrophic virus (HTLV), an oncogenic virus that is prevalent in indigenous women in the Peruvian Amazon, has been associated with HPV, suggesting the need for HTLV-positive women to be screened for HPV and cervical neoplasia 54. A low prevalence of HPV infection in four Amazon communities of Bolivia, where the majority of women tested were indigenous, suggests that other factors, including socioeconomic determinants, may play a role in cervical cancer genesis 55.

Cancer was associated with exposure to contaminants from oil drilling in the Amazon Basin of Ecuador, in a predominantly indigenous population 45,56, although the results are disputed 57 and a cluster of cancer cases among young indigenous people living near power lines in Pará, Brazil, was reported but we found no other reports in environmental exposure and cancer among indigenous people in LAC.

Two papers have reported protective factors for breast cancer, including breast feeding and a greater number of births, and less hormone replacement therapy and alcohol consumption, among selected communities of Indian women in Brazil 31,58, researchers suggesting that changing diet and increased rates of obesity may lead to an increased risk of breast cancer over time.

### Screening for cancer in indigenous people in LAC

Seven articles related to cancer screening and cytological programs among indigenous women in LAC were identified. Since 2005, a cervical screening program has been carried out among indigenous women in Xingu Indian Park, Brazil, with a reported coverage of 93%, and a decreased incidence in high-grade cervical lesions and invasive cancer reported in that population 59. A program in Guyana which targeted indigenous women (*n* = 2050), reported a high frequency of cervical cancer (without providing rates) and that 20% of women tested positive for HPV 47. A study of cervical cytology in indigenous women in Ecuador found evidence of dysplasia and recommended expanding coverage of cervical screening in that population 60 and a study of cervical cancer in French Guyana reported more aggressive lesions among Amerindian women 61.

Indigenous women reportedly have limited access to cervical screening services; 70% of indigenous women in Ecuador and 58% in a Guatemala report never having had a Pap smear 25, while indigenous women in Mexico report language difficulties and mistrust of the health system as barriers to participation in screening 62. A report regarding mammography and breast cancer among women in Mexico found that being indigenous increased the risk of not having a mammogram or follow-up after abnormal findings 63.

## Discussion

An appraisal of the peer-reviewed literature, cancer reports, and other sources revealed a paucity of data concerning cancer among indigenous people in LAC. Indeed, a 2006 comprehensive report on indigenous health in LAC makes no mention of cancer 5. This may reflect the precedence of competing health concerns such as malnutrition 64 and communicable diseases 65, but in view of projections that cancer will become a major cause of mortality worldwide in coming decades 66, and the regional evidence that cancer is emerging as a major public health issue in LAC 1, it is timely that the cancer burden is better measured in indigenous populations.

To the best of our knowledge, indigenous status is recorded in cancer registries of only four LAC countries and therefore there is no definitive way of quantifying and comprehending the cancer burden among indigenous peoples. In all, nine of the 35 papers identified for review were from Brazil, where less than 1% of the population are indigenous. With the exception of Bolivia (1983), no publication contained population-based cancer rates for indigenous people, and a number of reports highlight the lack of available data on cancer profiles of indigenous people 28,31,32. Cancer incidence rates, available for indigenous people for the first time from the PBCR in Brazil, were lower overall when compared with the total population but cancer survival is unknown.

The paucity of data aside, results point to cancer as an emerging public health problem among indigenous peoples in LAC over the next decades. Cervical cancer is the leading cause of death among women and, if current incidence rates remain unchanged, the burden of this cancer is predicted to increase by 75% in the region by 2025 67. Notably, cervical cancer rates tend to be elevated in those countries where a substantial proportion of the population are indigenous (Fig. 1), for example, in Bolivia, Haiti, Paraguay, Peru, El Salvador, and Nicaragua, and conversely, lowest in the Bahamas, Costa Rica, and Uruguay, where the indigenous populations comprise less than 15% of the total population. Despite the availability of screening services in most LAC countries, activities have largely been opportunistic 67, ineffective 60,61, and with limited access for rural women 68. The burden of gallbladder and stomach cancer, identified as cancers of high frequency among indigenous populations in Chile and other regions worldwide, is unreported for indigenous populations in other parts of LAC.

Empirical evidence suggests that improvements in social and economic status have led to an overall increase in life expectancy in Latin America 69, also predicted to increase the incidence of cancer in populations making the transition from low to higher levels of the Human Development Index (HDI) 66. Acculturation and ensuing increased rates of smoking 70, alcohol consumption 52, dietary changes, obesity 71, and environmental exposures 72 further expose indigenous people to cancer risk.

Indigenous communities are thus exposed to a double burden of cancer, one set of neoplasms related to westernized diet and lifestyle as communities transit from more traditional and isolated lifestyles, and a residual cancer burden linked with infections, pertaining more to poverty. This, coupled with a lack of access to health care services, including cancer screening and treatment, lower levels of education, and lower social status, contribute to poorer cancer survival among indigenous people. High-resource countries such as Australia 6, New Zealand 73, and the United States 74 have reported higher cancer mortality among indigenous populations, despite lower incidence rates in those populations.

A barrier to a critical assessment of cancer burden in indigenous populations is the lack of a universal definition of “indigenous” or “indigeneity,” although a generally accepted premise is that such peoples are the first inhabitants of a region or country and for whom existence predates colonization. The mechanism of identification used by countries and regions also varies, and may include self-identification, geographical concentration 75, use of an indigenous language, and phenotype 76; there is no single definition in LAC. Discrepancies also exist in the number of people identified as indigenous in some countries in LAC. The reasons are multiplicative and include inadequate or no enumeration of indigenous people in census records, and variation in methodologies where some enumeration includes only indigenous people who have no other racial mix in contrast to systems which recognize those with various ancestry, as long as there are indigenous antecedents. While inroads have been made in improving identification of indigenous people in Brazilian census records 77, the skin color/ethnicity variable is incomplete in approximately 5–55% cancer cases registered in PBCRs 33.

It is well recognized that robust cancer surveillance frameworks are necessary in order to develop, implement, and evaluate adequate cancer control programs 78,79. Without accurate population denominators, assessment of cancer rates will necessarily be imprecise, as has been noted for Native American populations in some regions of the United States 80. Very small populations of indigenous people present a further barrier to calculating cancer rates and it may take many years to obtain stable rates.

Poverty has been inextricably linked with poorer overall health 81,82 and poor cancer outcomes 83. In LAC, indigenous people suffer significantly worse socioeconomic conditions, exclusion from labor markets and have limited access to public education and health services compared to the population as a whole 2. Those in work earn 46–60% of the income of the nonindigenous population 16 and their history of exploitation and impoverishment has resulted in political and economic marginalization 84. Indigenous people have seen little of the same improvement in economic development that other people in Latin America have experienced over recent decades 11,21. Disparities in wealth (Table 2), education, and health are also correlated with an increased exposure to infections and subsequently risk of developing cancers of the liver hepatitis B virus (HBV) and hepatitis C virus (HCV), cervix (HPV), and stomach (*Helicobacter pylori*) 65.

**Table 2:** Population of indigenous people by country or region, with percentage of those living in poverty^1^

Country or region	Indigenous peoples (millions)	Indigenous poor (%)
China	106	5
South Asia	95	44
Southeast Asia	30	50
Africa	22	77
Arabia	15	7
**Central America and Mexico**	**12**	**75**
**South America**	**11**	**82**
Rest of world	9	22
Total	299	33

Latin American countries in bold.

1 As defined by Hall and Patrinos 95.

Health beliefs and systems for indigenous people in LAC range from traditional medicine practices 85 to a Western medical model, the first compromised by deforestation and other activities, and the second by inadequate access to mainstream health services, including vaccine and prevention programs 5. Indeed, cervical cancer screening programs are particularly limited for isolated inland populations of LAC 86, many of whom are indigenous, and access to histopathology or chemoradiation services are also limited 44,57. In addition, cultural inappropriateness of services, no health insurance, or health service unaffordability, as well as lack of proficiency in the language used within the health care system 2,5 are further barriers to screening and treatment. For example, participation in cervical screening programs in Bolivia was hindered when the majority of clients spoke an indigenous language and the care providers spoke Spanish 87.

Beliefs about cancer among some indigenous populations reportedly vary considerably from the biomedical model 88,89, but there is a paucity of information on the cancer beliefs of indigenous people in LAC 90, despite evidence that cancer care can be more effectively provided when there is a greater understanding and appreciation of diversity of beliefs 91.

Goss and colleagues rightly state that cancer registration in all countries in LAC must underpin cancer control measures, including prevention, screening, diagnosis, and treatment. The International Agency for Research on Cancer, through its Global Initiative (http://gicr.iarc.fr/) and roll-out of Regional Hubs for Cancer Registration in Latin America (as well as Africa and Asia), is well placed to assist countries in these efforts.
